# Computational design of treatment strategies for proactive therapy on atopic dermatitis using optimal control theory

**DOI:** 10.1098/rsta.2016.0285

**Published:** 2017-05-15

**Authors:** Panayiotis Christodoulides, Yoshito Hirata, Elisa Domínguez-Hüttinger, Simon G. Danby, Michael J. Cork, Hywel C. Williams, Kazuyuki Aihara, Reiko J. Tanaka

**Affiliations:** 1 Department of Bioengineering, Imperial College London, London SW7 2AZ, UK; 2 Institute of Industrial Science, University of Tokyo, Tokyo 153-8505, Japan; 3 Ecology Institute, National Autonomous University of Mexico, Mexico City 04510, Mexico; 4 School of Medicine and Biomedical Sciences, University of Sheffield, Sheffield, UK; 5 Centre of Evidence Based Dermatology, University of Nottingham, Nottingham, UK

**Keywords:** atopic dermatitis, treatment schedule, mathematical model, personalized treatment, optimal control theory, differential evolution

## Abstract

Atopic dermatitis (AD) is a common chronic skin disease characterized by recurrent skin inflammation and a weak skin barrier, and is known to be a precursor to other allergic diseases such as asthma. AD affects up to 25% of children worldwide and the incidence continues to rise. There is still uncertainty about the optimal treatment strategy in terms of choice of treatment, potency, duration and frequency. This study aims to develop a computational method to design optimal treatment strategies for the clinically recommended ‘proactive therapy’ for AD. Proactive therapy aims to prevent recurrent flares once the disease has been brought under initial control. Typically, this is done by using an anti-inflammatory treatment such as a potent topical corticosteroid intensively for a few weeks to ‘get control’, followed by intermittent weekly treatment to suppress subclinical inflammation to ‘keep control’. Using a hybrid mathematical model of AD pathogenesis that we recently proposed, we computationally derived the optimal treatment strategies for individual virtual patient cohorts, by recursively solving optimal control problems using a differential evolution algorithm. Our simulation results suggest that such an approach can inform the design of optimal individualized treatment schedules that include application of topical corticosteroids and emollients, based on the disease status of patients observed on their weekly hospital visits. We demonstrate the potential and the gaps of our approach to be applied to clinical settings.

This article is part of the themed issue ‘Mathematical methods in medicine: neuroscience, cardiology and pathology’.

## 1. Introduction

Atopic dermatitis (AD) is a chronic inflammatory disease, characterized by recurrent skin inflammation and a defective, permeable skin barrier, that is considered to be caused by complex interactions of genetic and environmental risk factors [1,2]. AD affects up to 25% of children worldwide and has associated socioeconomic burdens [3]. AD is associated with constant itching that may result in chronic sleep loss, and the resultant scratching can cause bleeding and skin infections. The current mainstay of AD treatment is to control the AD symptoms by topical application of corticosteroids or calcineurin inhibitors to reduce the inflammation, in addition to application of emollients to improve the barrier integrity [1,3,4]. However, only 24% of AD patients and carers believe that they adequately manage the symptoms by the current main treatments [5]. This is partly because clear guidance and consensus for effective treatment strategies in terms of frequency, duration and potency are yet to be fully established [2,3,6], while patients are often advised to minimize the use of corticosteroids because of a fear of skin thinning [7].

As a result of the complex underlying mechanisms of AD pathogenesis, AD patients demonstrate a wide spectrum of clinical phenotypes thereby greatly benefitting from personalized treatment [8]. Recently, long-term management of AD focuses on prevention of flares by the so-called ‘proactive therapy’ [9,10]. Following initial induction of remission to ‘get control’, proactive therapy aims to ‘keep control’ by preventing AD flares (inflammation) and achieving skin barrier stabilization. This is achieved by intermittent and scheduled use of low-dose topical corticosteroids or calcineurin inhibitors to the areas of the body that frequently have recurrent flares, even in the absence of the flares. Ideally, effective treatment schedules for proactive therapy can be tailored to each patient, based on the patient’s information, such as genetic risk factors, how the symptoms have evolved and the responses to the treatments. This paper investigates the potential of a computational method to inform the design of such personalized effective treatment schedules for proactive therapy, in terms of frequency, duration and potency.

We recently proposed a mathematical model of AD pathogenesis, as an *in silico* and quantitative framework that coherently explains underlying mechanisms of common AD phenotypes [11]. The model is a system-level representation of the complex and dynamic interplays between immune responses, skin barrier function and environmental triggers that determine the AD pathogenesis; specifically how AD flares start and how AD symptoms exacerbate. Our model simulations reproduced several sets of experimental and clinical results, providing plausible mechanistic and quantitative explanations for dynamic mechanisms behind onset, progression and prevention of AD.

In this study, we extend this experimentally validated mathematical model of AD pathogenesis and propose a new model of treatment effects on AD pathogenesis. We then use the mathematical model to computationally design the personalized optimal treatment schedules for proactive therapy by solving the optimal control problems recursively using a differential evolution (DE) algorithm [12,13], which is an efficient global optimization technique to solve our non-convex optimization problem.

## 2. Mathematical model of treatment effects on atopic dermatitis pathogenesis

**Figure 1.**
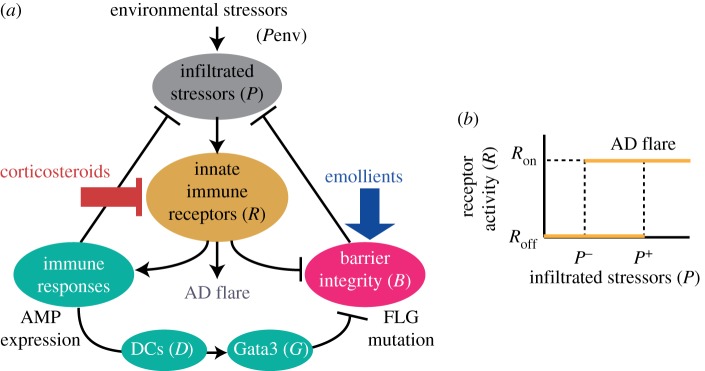
Mathematical model of treatment effects on AD pathogenesis. (*a*) A model schematic and (*b*) a perfect reversible switch for the AD flare triggered by infiltrated pathogens. The italics denote the model variables.

We consider a mathematical model of treatment effects on AD pathogenesis (figure 1*a*) obtained by incorporating the dynamic effects of treatments in the previous mathematical model of AD pathogenesis [11]. The proposed model is described by a set of three differential equations:
2.1
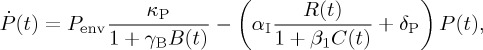

2.2
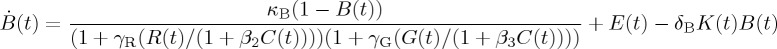

2.3
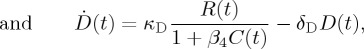
where *P*(*t*) is the amount of infiltrated environmental stressors, such as pathogens, that trigger the skin inflammation (AD flare) through activation of innate immune receptors (*R*(*t*)), *B*(*t*) denotes the strength of skin barrier integrity and *D*(*t*) denotes the level of inflammation markers, including pro-inflammatory cytokines (such as TSLP or IL33) and activated dendritic cells. A triple of the state variables, (*P*(*t*),*B*(*t*),*D*(*t*)), represents the patient’s disease status described by the levels of infection, barrier defects and inflammation. The variables *E*(*t*) and *C*(*t*), respectively, represent the potency of emollients and corticosteroids that are applied to achieve skin barrier stabilization and to prevent infection and inflammation. The parameters *β*_1_, *β*_2_, *β*_3_ and *β*_4_ represent the relative effects of corticosteroids on the relevant processes (table 1). Other model parameters and their nominal parameter values are taken from [11] (table 2).

**Table 1.** Description of simulation parameters and their nominal values.

symbol	name	nominal value
*β*_1_	corticosteroid-mediated rate of reduction of AMP expression	0.005
*β*_2_	corticosteroid-mediated rate of reduction in barrier damage	10
*β*_3_	corticosteroid-mediated rate of reduction of Th2 cytokine production	10
*β*_4_	corticosteroid-mediated rate of reduction of dendritic cell maturation	10
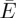	strength of emollients applied	0.04
*C*_max_	maximum strength of corticosteroids during the remission phase	50
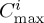	maximum strength of corticosteroids during the *i*th maintenance cycle	50
*T*^max^_r_	maximum duration of the induction phase	8 (weeks)
*T*_m_	duration of each maintenance cycle	1 (week)
*k*_1_	penalty weight for treatment duration	0.5
*k*_2_	penalty weight for total amount of treatment	1
*k*_3_	penalty weight for deviation of the final level of *P* from the target level	10
*k*_4_	penalty weight for deviation of the trajectories of *P* from the target level	10
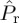	target level of *P* during the induction phase	24
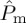	target level of *P* during the maintenance phase	26

**Table 2.** Description of model parameters and their nominal values.

symbol	name	nominal value
*P*_env_	environmental stress load	95 (mg ml^−1^)
*γ*_B_	barrier-mediated inhibition of pathogen infiltration	1
*κ*_P_	nominal skin permeability	0.85 (day^−1^)
*α*_I_	rate of pathogen eradication by innate immune responses	0.05 (day^−1^)
*δ*_P_	basal pathogen death rate	1.6 (day^−1^)
*κ*_B_	barrier production rate	0.5 (day^−1^)
*γ*_R_	innate immunity-mediated inhibition of barrier production	10
*δ*_B_	rate of kallikrein-dependent barrier degradation	0.1
*γ*_G_	adaptive immunity-mediated inhibition of barrier production	1
*κ*_D_	rate of DC activation by receptors	4 cells (ml×day)^−1^
*δ*_D_	rate of DC degradation	0.5 (day^−1^)
*P*^−^	receptor inactivation threshold	26.6 (mg ml^−1^)
*P*^+^	receptor activation threshold	40 (mg ml^−1^)
*R*_off_	receptor-off level	1
*R*_on_	receptor-on level	16.7

The proposed model describes the effects of the treatments on AD pathogenesis in a simple form, rather than explicitly incorporating the fine details of the complex molecular and cellular processes. The infiltrated stressors or pathogens, *P*(*t*), increase by the penetration of environmental stressors, *P*_env_, through the barrier, *B*(*t*). *P* is eradicated by innate immune responses triggered by inflammation (*R*(*t*)) and is also naturally degraded. Topical application of corticosteroids (*C*(*t*)) provides an anti-inflammatory action [4], resulting in a reduced antimicrobial protein (AMP) expression for eradication of pathogens [14]. The production of *B*(*t*) is described by a phenomenological representation of its capacity to self-restore the nominal barrier integrity (*B*=1) following its disruption, and is compromised by innate immune responses triggered by inflammation and by cytokines produced by differentiated Th2 cells (where the differentiation is controlled by the master transcription regulator Gata 3, *G*(*t*)). Topical application of corticosteroids reduces both inflammation and release of cytokines [3,15], resulting in improved skin barrier function [16]. Application of emollients enhances skin barrier integrity [3,4], helping reduce AD symptoms [17]. The degradation of the barrier occurs as a result of desquamation mediated by active kallikreins, *K*(*t*). The level of inflammation markers, *D*(*t*), increases while inflammation (*R*(*t*)) persists and degrades naturally.

The main aim of the proactive therapy is to prevent AD flares that are assumed to occur as a result of activation of the innate immune receptors. We model the AD flare by a perfect reversible switch between the off- and on-states (*R*=*R*_off_ and *R*_on_) with *P*^+^ and *P*^−^ denoting, respectively, the activation and inactivation thresholds for the infiltrated stressors (pathogens) that are recognized by the innate immune receptors (figure 1*b*) [11]. An AD flare occurs when the level of the stressors increases above *P*^+^, and stops when it decreases below *P*^−^. A similar switching behaviour is assumed for *K* [11].

In our study, we consider moderate to severe AD patients, who could benefit from proactive therapy for flare prevention and skin barrier stabilization. The severity of the AD symptoms is characterized in our model by two model parameters, *κ*_P_ and *α*_I_, that correspond to the level of the genetic risk factors: mutations in the FLG gene (*κ*_P_) and a dysregulated expression of innate immune system components (*α*_I_) including regulators of antimicrobial peptide expression (e.g. TLRs and NF-*κ*B) [11,18]. A lower *α*_I_ indicates dysfunctional immune responses and a diminished capacity to eradicate the infiltrating pathogens, and a higher *κ*_P_ results in a higher skin barrier permeability leading to increased infiltration of stressors that can cause the recurrence of AD flares. To simulate these moderate to severe AD patients, we pick the values of (*α*_I_,*κ*_P_) from the parameter region for which a unique pathological steady state exists that corresponds to the high level of stressors leading to a persistent AD flare and completely damaged skin barrier. We also assume *R*_off_=1 and *K*_off_=1 to model the effects of subclinical inflammation in non-lesional skin of severe AD patients [10], and *G*(*t*)=1 corresponding to systemic Th2 sensitization due to the persistent AD flare [11].

## 3. Optimal control problem formulation

**Figure 2.**
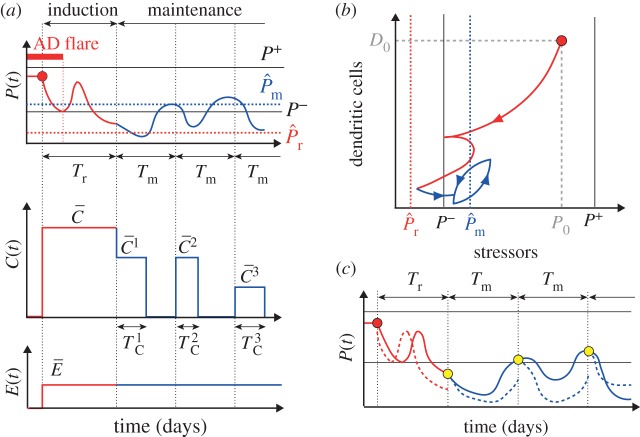
Optimal control problem formulation to design treatment strategies for proactive therapy for AD. The whole period consists of the induction phase (red) and maintenance phase (blue). (*a*) An example dynamics of a state variable (*P*(*t*)) with on–off treatment of corticosteroids (*C*(*t*)) of different potencies at discrete times, in addition to constant application of emollients (*E*(*t*)) of a fixed potency 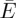. (*b*) An example trajectory of a projection of a patient’s states, (*P*,*D*), when the optimal treatment strategy is applied. The states move from the initial state ((*P*_0_,*D*_0_), red circle) towards the target level, 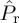, of the induction phase (red vertical line), and then to the target level, 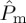, of the maintenance phase (blue vertical line). The state *B* is omitted in the figure. (*c*) The recursive optimal control problem to be solved. The optimal treatment strategy for each cycle (either for the duration of *T*_r_ or *T*_m_) is determined by predicting the optimal evolution of the state variables (dashed lines) based on the measurement of the states at the beginning of the period (circles). The actual evolution of the state (solid lines) can be different from the prediction, for example if the calculated optimal treatment strategy is not applied.

Using the proposed model, we computationally design optimal treatment strategies for proactive therapy with a combination of emollients and corticosteroids. The proactive therapy consists of two phases: an ‘induction of remission’ phase where we aim to suppress the clinical inflammation, followed by a ‘maintenance of remission’ phase where we apply intermittent but scheduled treatment to prevent the recurrence of the AD flare [9,10]. We refer to the two phases as ‘induction phase’ and ‘maintenance phase’ hereafter. The induction phase aims to ‘get control’, and the maintenance phase aims to ‘keep control’ [10]. To comply with the current clinical recommendations [3,19], we assume that emollients are applied constantly throughout both phases at a low level, 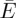, which is insufficient to achieve the remission by itself for moderate to severe AD patients [20]. We, therefore, design the optimal schedules for application of corticosteroids that can induce and maintain the remission. We consider the on–off treatment at discrete times, reflecting the daily application or non-application of corticosteroids with different potencies (figure 2*a*).

We formulate the problem as finding optimal treatment strategies that minimize the duration and potency of the treatments that effectively move the state variables from the initial steady state to the specified target state, while the dynamics of the state variables is determined by our model (figure 2*b*). The target state is defined by a target level of *P*(*t*) that does not lead to recurrence of an AD flare (figure 1*b*), as the proactive therapy aims to prevent the AD flare. The objective function, *J*, is described by *J*=*k*_1_*J*_1_+*k*_2_*J*_2_+*k*_3_*J*_3_+*k*_4_*J*_4_, where *J*_1_ is the penalty for the treatment duration, corresponding to the patients’ burden to apply the treatments, *J*_2_ is the penalty for the total amount of the treatment applied (duration × strength), representing the financial cost as well as the risk of side effects due to the excessive use of corticosteroids, *J*_3_ is the penalty for the final state to be deviated from the target state and *J*_4_ is the penalty for the trajectory to be deviated from the target state. The functions *J*_1_, *J*_2_, *J*_3_ and *J*_4_ are defined for the induction and maintenance phases as shown below. We assume *k*_3_=*k*_4_ (the coefficients for the penalty on the deviation from the target state) for simplicity.

### (a) Induction of remission phase

In the induction phase, we assume that corticosteroids are constantly applied during the calculated optimal duration, *T*_r_. The optimal problem to be solved is formulated so as to find a pair of values, (*T*_r_, 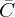), where *T*_r_ is the duration of the induction phase and 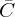 is the potency of corticosteroid to be constantly applied during this phase to minimize the objective function under the constraints 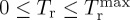 and 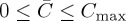. We set the target level, 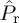, to be smaller than *P*^−^ where the AD flare ceases. The objective function for the induction phase consists of 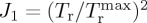, 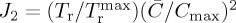, 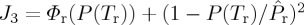 and 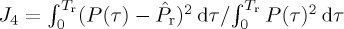. *Φ*_r_(*P*(*T*_r_)) is a non-convex function that penalizes the failure to achieve remission and takes the values of 100+0.1(*P*(*T*_r_)−*P*^−^) if *P*(*T*_r_)>*P*^−^ and 0 otherwise.

### (b) Maintenance of remission phase

Once the remission is induced, the proactive therapy proceeds to the maintenance phase, where corticosteroids are applied intermittently to prevent the recurrence of AD flares. We design the optimal treatment schedule for a week, based on the measurement of the state variables at the beginning of the week, and repeat the weekly cycle (figure 2*c*). This scenario corresponds to weekly hospital visits of the patients where clinicians evaluate the disease state and plan the treatment strategy until the next visit.

For the *i*th cycle of the maintenance phase, we calculate the optimal treatment strategy (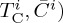 that minimizes the objective function, under the constraints on the duration of corticosteroid application, 0≤*T*^*i*^_C_≤*T*_m_=7 days, and its potency 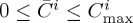. For the whole duration of the maintenance phase, we set the target level, 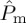, that is smaller than *P*^+^ to avoid the recurrence of the AD flare (figure 1*b*). In the next section, we investigate the effects of the choice of 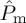 on the calculated optimal treatment strategies. The objective function for the *i*th maintenance cycle consists of *J*_1_=(*T*^*i*^_C_/*T*_*m*_)^2^, 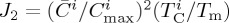, 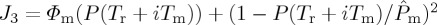 and 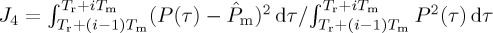. A non-convex function, *Φ*_m_(*P*(*T*_r_+*iT*_m_)), represents the penalty on the re-occurrence of an AD flare during the maintenance phase, and takes the values of 100+0.1(*P*(*T*_r_+*iT*_m_)−*P*^−^) if both *P*(*T*_r_+*iT*_m_)>*P*^−^ and *R*(*t*)=*R*_on_ are satisfied, and 0 otherwise.

## 4. Computational identification of optimal treatment schedules for moderate to severe atopic dermatitis patients

**Figure 3.**
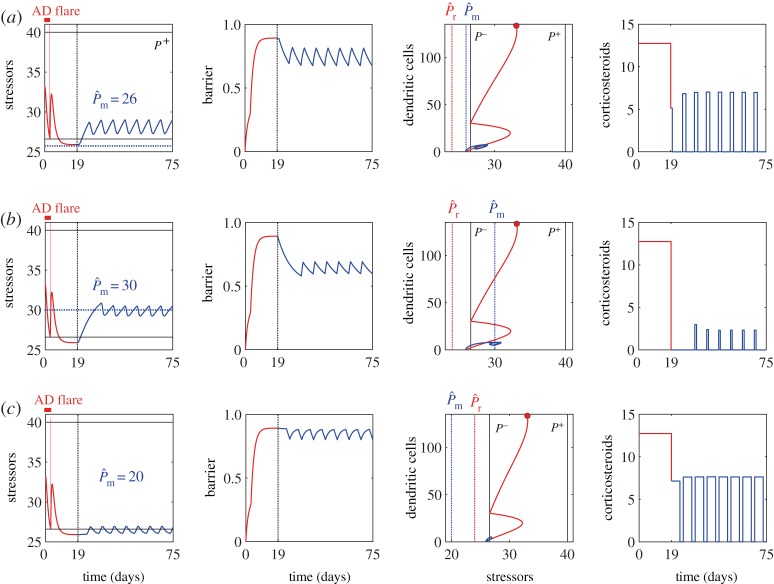
Effects of the choice of the maintenance target level (blue dotted lines) on the optimal treatment strategies calculated and the resulting dynamics of the system. The maintenance target level is set to be (*a*) 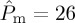 (the nominal value), (*b*) 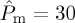 and (*c*) 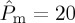, while the induction target level (red vertical lines) is set to be the same (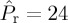) for all the scenarios. The dynamics during the induction phase and that during the maintenance phase are shown in red and blue, respectively. Remission is achieved when the level of the stressors is decreased below *P*^−^, and the AD flare reoccurs when it increases above *P*^+^. The induction phase continues even after the AD flare ceases, and tries to bring the state towards the target level.

We used DE to solve the optimal control problem formulated above, for different scenarios, to test the applicability of our approach. The nominal values used for the simulations for moderate to severe AD patient cohorts, such as the target levels and the constraints on the strength and duration of treatments, are summarized in table 1. We first confirmed that the optimal treatment strategy calculated using the nominal parameter values suggests a length of the induction phase, *T*_*r*_, that is clinically relevant (less than or equal to four weeks [21]). Indeed, the optimal induction period was calculated to be *T*_r_=19 days, while the AD flare stopped within the first 3 days (figure 3*a*). We also confirmed by global sensitivity analysis that this calculated optimal strategy is robust to changes of the model parameters and choice of the weighting coefficients for the objective function (electronic supplementary material, figures S1 and S2).

### (a) Effects of the choice of the maintenance target level

We investigated the effects of the choice of the target level during the maintenance phase on the calculated optimal treatment strategies (figure 3).

In the nominal case with the nominal parameter values, we set the maintenance target level to be 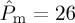, which is lower than the deactivation threshold (*P*^−^=26.6) but higher than the induction target level (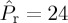). The calculated optimal maintenance treatment (figure 3*a*) demonstrates that intermittent application of corticosteroids by 3 days per week could achieve the maintenance without recurrence of the AD flare for the whole duration of the maintenance phase investigated (eight weeks).

When the maintenance target is chosen to be closer to the activation threshold (*P*^+^=40), for example 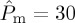, the calculated optimal treatment strategy suggests the application of corticosteroids by 1 day per week to maintain the remission (figure 3*b*). This higher target level is much easier to be maintained with a smaller amount of corticosteroids. However, the resulting state corresponds to a lower barrier integrity with a higher level of infiltrated stressors, compared with the nominal scenario (figure 3*a*), due to a smaller amount of corticosteroid applied. This worsening of the state may make patients more vulnerable to an increased level of environmental stressors due to random or natural fluctuations that can retrigger an AD flare.

On the contrary, when the maintenance target is chosen to be further lower than *P*^−^, for example 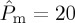, the optimal treatment strategy requires an increased amount of corticosteroids, with application of more potent corticosteroids for 6 days per week, to achieve a very low target value (figure 3*c*). While the calculated optimal treatment strategy can successfully prevent recurrence of inflammation, this strategy is not desired due to the high amount of corticosteroids applied in total (2.7-fold increase from the nominal case).

These results suggest the importance of the choice of the maintenance target level, 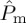, as a design criterion for optimal treatment strategies. We decided to use 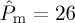 for further simulations, as it ensures the successful maintenance of remission without the need of excessive treatment amount during the maintenance phase and it may correspond to the clinically suggested so-called weekend therapy.

We also investigated the effects of choice of the induction target, 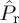 (figure not shown). Decrease of 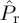 from our nominal value of 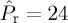 resulted in an optimal strategy that requires an increased amount of corticosteroid (in both the potency and application time) during the induction phase, but did not affect the strategy during the maintenance phase.

### (b) Stratification of patient cohorts

To demonstrate that our approach is applicable to different patient cohorts, we computationally obtained the optimal treatment strategies for different values of two model parameters, (*κ*_*P*_,*α*_I_), that can specify patient cohorts by their strengths of the two main genetic risk factors, mutations in the FLG gene (*κ*_P_) and a dysregulated expression of innate immune system components (*α*_I_). The objective functions and target states were the same as the nominal case.

**Figure 4.**
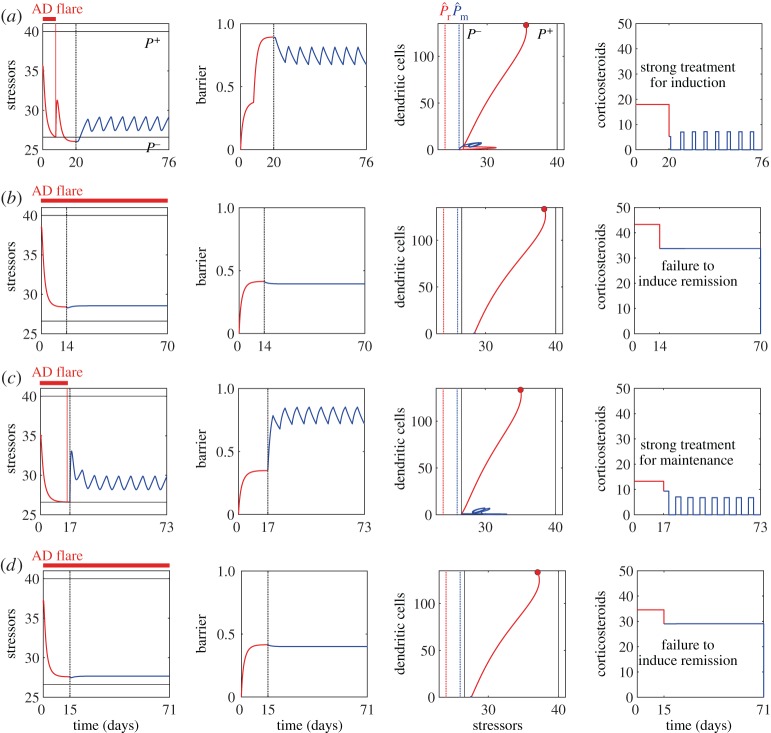
Calculated optimal treatment strategies for different patient cohorts with dysfunctional immune responses: (*a*) (*κ*_P_,*α*_I_)=(0.85,0.04) and (*b*) (*κ*_P_,*α*_I_)= (0.85,0.03); and compromised barrier integrity: (*c*) (*κ*_P_,*α*_I_)=(0.9,0.05) and (*d*) (*κ*_P_,*α*_I_)=(0.95,0.05). The dynamics during the induction phase starting from the initial state (red circles) is shown in red, and that for the maintenance phase is in blue.

Specifically, we investigated three patient cohorts: the nominal patient cohorts with (*κ*_P_,*α*_*I*_)=(0.85,0.05) (figure 3*a*), those with even more dysfunctional immune responses described by (*κ*_P_,*α*_I_)=(0.85,0.04) (figure 4*a*) and (*κ*_P_,*α*_I_)=(0.85,0.03) (figure 4*b*), and those with even more compromised barrier integrity described by (*κ*_*P*_,*α*_I_)=(0.9,0.05) (figure 4*c*) and (*κ*_P_,*α*_I_)=(0.95,0.05) (figure 4*d*). All the parameters, except for *κ*_P_ and *α*_I_, were set to be the nominal values (tables 1 and 2).

For the case with (*κ*_P_,*α*_I_)=(0.85,0.04) (figure 4*a*), the calculated optimal treatment strategy was successful in achieving remission and preventing AD flares for eight consecutive weeks, by an induction treatment of 20 days followed by 3 days per week maintenance treatment. However, it requires a higher amount of corticosteroids (48% increase for the induction phase and 3.3% increase for the maintenance phase) of a higher potency, compared to the nominal case (figure 3*a*), to combat the higher initial stressor load. When the risk factor of dysfunctional immune responses becomes even stronger, as for the case with (*κ*_P_,*α*_I_)=(0.85,0.03) (figure 4*b*), the calculated optimal treatment schedule failed to achieve remission, leading to sustained or unresolved AD symptoms with a low barrier integrity. Indeed, the ‘maintenance’ therapy we computationally calculated after 14 days of the ‘failed’ induction of remission suggests a continuous use of corticosteroids, meaning that these virtual patients will require constant, rather than intermittent, application of corticosteroids (figure 4*b*).

For the cohorts with an increased barrier permeability (*κ*_P_=0.9), an increased amount of corticosteroid (by 54.7%) is required during the maintenance phase (figure 4*c*), compared to the nominal cohort with the same *α*_*I*_=0.05. Further increase in the barrier permeability (*κ*_P_=0.95) led to failure in inducing remission and the optimal strategy suggests to continue the constant application of corticosteroids (figure 4*d*). Synergistic effects of the two risk factors, by increasing *κ*_P_ and decreasing *α*_I_ simultaneously ((*κ*_*P*_,*α*_I_)=(0.9,0.04), figure not shown), resulted in an optimal strategy with a 5.8-fold increase in the total amount of corticosteroid (2.35-fold in the induction phase and 11.3-fold in the maintenance phase), compared to the nominal cohort. However, the optimal strategy still could not achieve remission.

These results suggest that our approach is applicable to different virtual patient cohorts, and that it could help stratify the virtual patients into those who would benefit from the calculated optimal treatment strategies, and those who would require additional or stronger treatments, such as systemic treatment, to achieve remission. Our results also demonstrate how the treatment efforts scale with the level of the two common AD risk factors. It will be interesting to compare the computational predictions with the patients’ data that relate the severity of the patients’ symptoms to the required treatments and the actual treatments prescribed. Evaluation of how the treatment efforts scale with the initial disease severity before the start of treatments in the clinic is an interesting future research topic.

### (c) Effects of poor adherence to suggested optimal treatment schedule

So far, we assumed that the patients follow the calculated optimal treatment schedule. However, AD patients do not necessarily always follow the treatment guidelines. This problem of poor adherence to treatment could have negative effects on long-term treatment of AD [22,23]. Using our proposed approach, we investigated the effects of poor adherence to the calculated optimal treatment schedule, particularly how the future optimal treatment schedule and the evolution of the disease state are affected.

**Figure 5.**
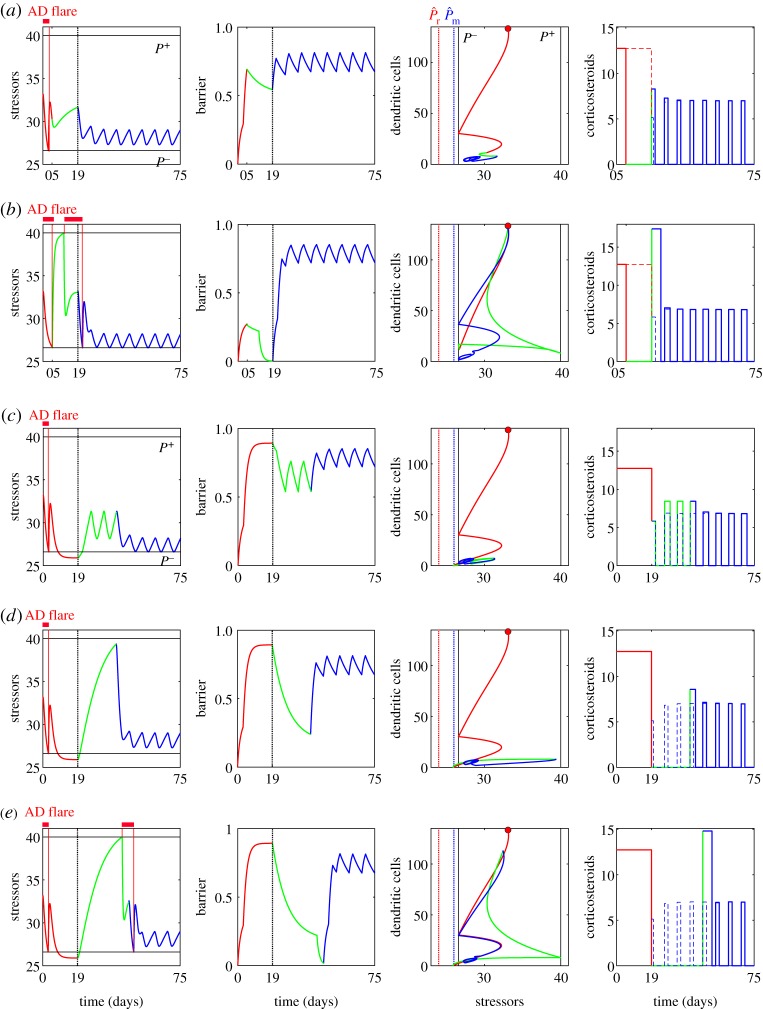
Effects of poor adherence to the calculated optimal treatment strategies. Non-adherence to the suggested corticosteroid treatment after 5 days in the induction phase, with continual use of emollients (*a*) and without use of emollients (*b*). Non-adherence to the suggested corticosteroid treatment during the maintenance phase, without emollient application for the first three cycles (*c*), and without both corticosteroid and emollient application for the first three cycles (*d*) and four cycles (*e*). Green lines represent the period during the non-adherence, and the dotted lines on the right column demonstrate the optimal strategy calculated for the nominal case where patients adhere to the suggested optimal strategy.

Consider the case where the virtual patients do not complete the calculated optimal induction phase and stop using corticosteroids after 5 days when the AD flare disappears. This scenario can occur, for example, due to corticosteroid phobia. If they continue their daily emollient treatment (figure 5*a*), the optimal strategy in the subsequent weeks suggests the use of an increased amount of corticosteroid, with a 1.4-fold more potent corticosteroid for a longer period (by 1 day) during the first maintenance cycle, compared with the nominal case. The effect of non-adherence is not predicted to be dramatic, possibly because the AD flare ceased already within the first 5 days of corticosteroid use. However, if the virtual patients also stop the daily emollient treatment (figure 5*b*), we observe the recurrence of an AD flare with a severe worsening of the symptoms (a sharp decrease in *B* and a dramatic increase in *P* and *D*). As a result, the calculated optimal strategy in the subsequent cycle suggests the use of a more potent corticosteroid (by 1.4-fold) than that applied during the induction phase, for 5 days. This corresponds to a prolonged induction phase with a more potent corticosteroid. If we prolong the maintenance cycle to 20 days from 7 days, the calculated optimal strategy suggests the application of a corticosteroid of 10% more potent than that used for the induction phase, for an extra duration of 10 days (figure not shown). These computational predictions suggest the benefits of continual use of emollients in reducing AD symptoms, as shown in [3].

Another scenario to be considered is the non-adherence during the maintenance phase, following the successful completion of the induction phase. When the virtual patients stop their daily application of emollients for the first three maintenance cycles (figure 5*c*), it results in an immediate worsening of the symptoms (a decrease in *B*). The calculated optimal strategy suggests application of more potent corticosteroids (by 1.4-fold) than the nominal case for 3 days per week in the subsequent three weeks to maintain the remission. When they also stop applying corticosteroids during these three weeks (figure 5*d*), a severe worsening of the symptoms (a sharp decrease in *B* and a dramatic increase in *P* and *D*) is observed. The calculated optimal strategy then suggests application of an increased amount of corticosteroid (1.5-fold more potent for two more days) than the nominal case to maintain the remission in the subsequent weeks. If they miss the treatments for four weeks (figure 5*e*), the AD flare reoccurs and the optimal strategy suggests the use of a much more potent corticosteroid (by 1.35-fold) than that used during the induction phase, for the duration of 5 days.

## 5. Discussion

In this paper, we proposed a computational method to inform the design of patient-specific optimal treatment strategies for moderate to severe AD patients who require constant treatment for stabilization of their AD symptoms. Our proposed framework solves optimal control problems recursively to design treatment schedules for proactive therapy that aims to prevent AD flares and to achieve skin barrier stabilization. The proactive therapy consists of intermittent and scheduled use of low-dose corticosteroids, in addition to a constant application of emollients, once initial induction of remission has been achieved. The objective functions to be minimized correspond to the penalties on the duration and the potency of the treatments applied, as well as the deviation from the target states we specify.

One of the main difficulties in formulating an optimal control problem is to identify appropriate objective functions. Here, we systematically explored different possible target levels for the clinically relevant variables to be controlled (*P*) and found the most adequate maintenance target level (figure 3), which we used to derive robust treatment strategies even with poor adherence (figure 5), for our nominal patient cohorts and for patient cohorts with severer genetic risk factors (figure 4). These results suggest the importance of choosing appropriate target states to successfully maintain remission, and that our proposed mathematical framework can be used to investigate the effects of poor adherence to the optimal treatment strategies systematically. We could also identify those virtual patient cohorts that would require stronger treatments, with even higher doses or additional pharmacological substances such as antibiotics, phototherapy or systemic immunosuppressant treatment to achieve adequate disease control (figure 4). We evaluated the sensitivity of our results to the changes in the values of *k*_1_, *k*_2_, *k*_3_ and *k*_4_, and the model parameters, and confirmed that the optimal strategies calculated for the nominal case are robust (electronic supplementary material, figures S1 and S2).

Our systematic and computational approach could be effective in informing the design of personalized optimal treatment strategies, and help to solve the issues with the current lack of a clear guidance and consensus for effective treatment strategies, and to minimize the potential side effects of long-term use of corticosteroids. Moderate to severe AD patients require repeated treatment to stabilize their pathological state that would naturally remain unresolved if treatment is not applied. In addition, moderate to severe AD patients usually require a combination of treatments (such as corticosteroids and emollients) to be applied to achieve successful maintenance of remission in two phases (induction and maintenance phases). Accordingly, designing the optimal treatment strategies to stabilize the AD symptoms for these patients may benefit from an advanced optimization technique such as the one proposed in this paper.

In this paper, we computationally obtained the optimal treatment strategy by recursively solving the optimal control problems. The proposed computational framework could be easily extended to the application of model predictive control (MPC), which uses the measured states of the system to predict and optimize the control input that minimizes the objective function over a future time horizon [24]. MPC has been already successfully applied to design treatment profiles for diabetes [25,26], prostate cancer [27] and leukaemia [28,29]. Application of MPC, i.e. inclusion of the receding horizon, will allow us to obtain smoother graded therapy, as it will enable us to find the optimal treatment schedule that does not necessarily achieve the target level within each maintenance cycle but achieves it gradually over a longer period.

Our simulation results in this paper show that poor adherence to the suggested optimal treatment schedule inevitably leads to higher treatment doses in subsequent cycles. This indicates a potential benefit of using our approach under more realistic clinical scenarios to provide a theoretical argument to recommend patients to adhere to the suggested treatment strategies. Our results are also consistent with the current clinical recommendations, for example, the weekend therapy with application of corticosteroids for two consecutive days per week, in addition to the daily application of emollients. Our investigation on the effects of the AD flare that occurs during maintenance therapy demonstrated that resuming continuous use of a much stronger corticosteroid can successfully achieve remission but result in an increase in the total amount of corticosteroids applied. As an important next step, we need to compare the computationally obtained treatment strategies with treatment options that are currently used in the clinical setting.

We also need to develop robust ways to identify the simulation parameters and the model parameters from each patient’s clinical data such as initial skin thickness and pattern of eczema, in order to effectively calculate the optimal treatment strategies. If the temporal data of patients become available, the information on the discrepancy between the measured values and the model prediction will be used to identify the model parameters online. The approach we proposed in this paper could be a first step towards designing personalized effective treatment strategies for prevention, and adequate control, of AD symptoms. Exploring the personalized optimal treatment strategies in the clinical setting would be challenging if we do not apply a systematic and computational approach, because of the combinatorial explosion of treatment types, durations, potencies and each patient’s information. As the model was developed based on the understanding of the pathological mechanisms, the obtained treatment strategies could be readily interpreted and the framework is applicable to different patient cohorts and different scenarios. For example, it will help to identify a way to reduce the frequency of clinic visits by placing control back in the hands of parents and children, evaluate the effects of reduced visits and whether we can still achieve the optimal treatment strategies by monthly or bimonthly clinical visits.

This paper demonstrated the proof of concept of the computational design of optimal treatment strategies, using a mathematical model that describes the treatment effects in a simple form. We will further investigate the appropriateness of the model description of the treatment effects, using dynamic data of AD patients after application of corticosteroids and emollients.

## 6. Material and methods

All simulations were conducted using Matlab v. R2016a (The MathWorks, Inc., Natick, MA, USA). We used *ode45* for the numerical integration of the system and *events location* functionality of Matlab to identify the switching boundaries of the hybrid system. We identified the optimal treatment strategy for the induction phase by applying a DE algorithm with 1000 generations. Starting from 30 randomly chosen initial vectors, (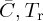), with 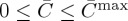 and 0≤*T*_*r*_≤*T*^max^_r_, we found the optimal solution by evolving a population of 30 vectors at each generation, using the mutation strategy DE/rand-to-best/1 [30] with a differential weight of 0.6 and recombination with a crossover rate of 0.5. The same procedures were repeated for each maintenance cycle. The global sensitivity analysis was conducted by simultaneously varying the values of the model parameters or the weights for the objective functions from their nominal values by ±50% for 529 and 400 simulations, respectively.

## Supplementary Material

Supplementary material

## Data accessibility

All the necessary pieces of information to reproduce this study are included in this paper and its supplementary material available online.

## References

[1] Weidinger S, Novak N 2016 Atopic dermatitis. *Lancet* 387, 1109–1122. (10.1016/S0140-6736(15)00149-X) 26377142

[2] Bieber T *et al.* 2016 Global Allergy Forum and 3rd Davos Declaration 2015: atopic dermatitis/eczema: challenges and opportunities toward precision medicine. *Allergy* 71, 588–592. (10.1111/all.12857) 27023268

[3] Eichenfield LF *et al.* 2014 Guidelines of care for the management of atopic dermatitis. *J. Am. Acad. Dermatol.* 71, 116–132. (10.1016/j.jaad.2014.03.023) 24813302 PMC4326095

[4] Simpson EL 2010 Atopic dermatitis: a review of topical treatment options. *Curr. Med. Res. Opin.* 26, 633–640. (10.1185/03007990903512156) 20070141

[5] Zuberbier T *et al.* 2006 Patient perspectives on the management of atopic dermatitis. *J. Allergy Clin. Immunol.* 118, 226–232. (10.1016/j.jaci.2006.02.031) 16815160

[6] Williams HC 2005 Atopic dermatitis. *N. Engl. J. Med.* 352, 2314–2324. (10.1056/NEJMcp042803) 15930422

[7] Cork MJ *et al.* 2009 Epidermal barrier dysfunction in atopic dermatitis. *J. Invest. Dermatol.* 129, 1892–1908. (10.1038/jid.2009.133) 19494826

[8] Bieber T 2015 Personalized management of atopic dermatitis: beyond emollients and topical steroids. In Personalized treatment options in dermatology , pp. 61–76. Berlin, Germany: Springer. (10.1007/978-3-662-45840-2).

[9] Wollenberg A, Ehmann LM 2012 Long term treatment concepts and proactive therapy for atopic eczema. *Ann. Dermatol.* 24, 253–260. (10.5021/ad.2012.24.3.253) 22879707 PMC3412232

[10] Tang TS, Bieber T, Williams HC 2014 Are the concepts of induction of remission and treatment of subclinical inflammation in atopic dermatitis clinically useful? *J. Allergy Clin. Immunol.* 133, 1615–1625. (10.1016/j.jaci.2013.12.1079) 24655575

[11] Domínguez-Hüttinger E, Christodoulides P, Miyauchi K, Irvine AD, Okada-Hatakeyama M, Kubo M, Tanaka RJ 2017 Mathematical modelling of atopic dermatitis reveals ‘double switch’ mechanisms underlying 4 common disease phenotypes. *J. Allergy Clin. Immunol.* 139, 31 433–31 436. (10.1016/j.jaci.2016.10.026) 27931974

[12] Storn R, Price K 1997 Differential evolution—a simple and efficient heuristic for global optimization over continuous spaces. *J. Glob. Optim.* 11, 341–359. (10.1023/A:1008202821328)

[13] Price KV, Storn RM, Lampinen JA 2005 *Differential evolution*. Natural Computing Series Berlin, Germany: Springer (10.1007/3-540-31306-0)

[14] Jensen JM *et al.* 2011 Differential suppression of epidermal antimicrobial protein expression in atopic dermatitis and in EFAD mice by pimecrolimus compared to corticosteroids. *Exp. Dermatol.* 20, 783–788. (10.1111/j.1600-0625.2011.01322.x) 21707760

[15] Rozkova D, Horvath R, Bartunkova J, Spisek R 2006 Glucocorticoids severely impair differentiation and antigen presenting function of dendritic cells despite upregulation of Toll-like receptors. *Clin. Immunol.* 120, 260–271. (10.1016/j.clim.2006.04.567) 16765091

[16] Jensen JM, Pfeiffer S, Witt M, Bräutigam M, Neumann C, Weichenthal M, Schwarz T, Fölster-Holst R, Proksch E 2009 Different effects of pimecrolimus and betamethasone on the skin barrier in patients with atopic dermatitis. *J. Allergy Clin. Immunol.* 123, 1124–1133. (10.1016/j.jaci.2009.03.032) 19410693

[17] Mason JM, Carr J, Buckley C, Hewitt S, Berry P, Taylor J, Cork MJ 2013 Improved emollient use reduces atopic eczema symptoms and is cost neutral in infants: before-and-after evaluation of a multifaceted educational support programme. *BMC Dermatol.* 13, 7 (10.1186/1471-5945-13-7) 23679991 PMC3665665

[18] Domínguez-Hüttinger E, Ono M, Barahona M, Tanaka RJ 2013 Risk factor-dependent dynamics of atopic dermatitis: modelling multi-scale regulation of epithelium homeostasis. *Interface Focus* 3, 20120090 (10.1098/rsfs.2012.0090) 23853706 PMC3638487

[19] Ring J *et al.* 2012 Guidelines for treatment of atopic eczema (atopic dermatitis). II. *J. Eur. Acad. Dermatol. Venereol.* 26, 1176–1193. (10.1111/j.1468-3083.2012.04636.x) 22813359

[20] Ng SY, Begum S, Chong SY 2016 Does order of application of emollient and topical corticosteroids make a difference in the severity of atopic eczema in children? *Pediatr. Dermatol.* 33, 160–164. (10.1111/pde.12758) 26856694

[21] Schmitt J, von Kobyletzki L, Svensson A, Apfelbacher C 2011 Efficacy and tolerability of proactive treatment with topical corticosteroids and calcineurin inhibitors for atopic eczema: systematic review and meta-analysis of randomized controlled trials. *Br. J. Dermatol.* 164, 415–428. (10.1111/j.1365-2133.2010.10030.x) 20819086

[22] Aubert-Wastiaux H *et al.* 2011 Topical corticosteroid phobia in atopic dermatitis: a study of its nature, origins and frequency. *Br. J. Dermatol.* 165, 808–814. (10.1111/j.1365-2133.2011.10449.x) 21671892

[23] Ellis RM, Koch LH, McGuire E, Williams JV 2011 Potential barriers to adherence in pediatric dermatology. *Pediatr. Dermatol.* 28, 242–244. (10.1111/j.1525-1470.2011.01493.x) 21615470

[24] Camacho EF, Bordons C 2007 Model predictive control . Advanced Textbooks in Control and Signal Processing. London, UK: Springer. (10.1007/978-0-85729-398-5).

[25] Clarke WL, Anderson S, Breton M, Patek S, Kashmer L, Kovatchev B 2009 Closed-loop artificial pancreas using subcutaneous glucose sensing and insulin delivery and a model predictive control algorithm: the Virginia experience. *J. Diabetes Sci. Technol.* 3, 1031–1038. (10.1177/193229680900300506) 20144416 PMC2769907

[26] Hovorka R *et al.* 2010 Manual closed-loop insulin delivery in children and adolescents with type 1 diabetes: a phase 2 randomised crossover trial. *Lancet* 375, 743–751. (10.1016/S0140-6736(09)61998-X) 20138357

[27] Hirata Y, Azuma S, Aihara K 2014 Model predictive control for optimally scheduling intermittent androgen suppression of prostate cancer. *Methods* 67, 278–281. (10.1016/j.ymeth.2014.03.018) 24680737

[28] Noble SL, Sherer E, Hannemann RE, Ramkrishna D, Vik T, Rundell AE 2010 Using adaptive model predictive control to customize maintenance therapy chemotherapeutic dosing for childhood acute lymphoblastic leukemia. *J. Theor. Biol.* 264, 990–1002. (10.1016/j.jtbi.2010.01.031) 20138060

[29] Jayachandran D, Rundell AE, Hannemann RE, Vik TA, Ramkrishna D 2014 Optimal chemotherapy for leukemia: a model-based strategy for individualized treatment. *PLoS ONE* 9, e109623 (10.1371/journal.pone.0109623) 25310465 PMC4195683

[30] Yang M, Li C, Cai Z, Guan J 2015 Differential evolution with auto-enhanced population diversity. *IEEE Trans. Cybern.* 45, 302–315. (10.1109/TCYB.2014.2339495) 25095277

