# Supplementary material for: Computational design of treatment strategies for proactive therapy on atopic dermatitis using optimal control theory
Source: Philos Trans A Math Phys Eng Sci. 2017 May 15;375(2096):20160285. doi: 10.1098/rsta.2016.0285 (PMC5434076; doi:10.1098/rsta.2016.0285)
Supplement: Supplementary material [file rsta20160285supp1.pdf]

Supplementary material: Computational design of treatment strategies for proactive therapy on atopic dermatitis using optimal control theory

P. Christodoulides, Y. Hirata, E. Domínguez-Hüttinger, S. G. Danby,

M. J. Cork, H. C. Williams, K. Aihara, and R. J. Tanaka *

We conducted sensitivity analysis to confirm that the optimal strategies calculated for the nominal parameter set (Tables 1 and 2) are robust to changes in the model parameters and the weighting coefficients for the objective function. The robustness was evaluated by whether

- the duration of the optimal induction phase (T_{r}) corresponds to the clinically accepted range of less than 4 weeks,
- the optimal treatment duration in each maintenance cycle (T_{C}^{i}) corresponds to 2-3 days (weekend therapy), and
- the potency of corticosteroid required during the maintenance phase is lower than that required during the remission phase ($\bar{C} > \bar{C}^{\mathrm{i}}$).

1 Sensitivity to risk factors $(\kappa_{\mathrm{P}}, \alpha_{\mathrm{I}})$

We calculated the optimal treatment strategies for $N = 1600$ combinations of $(\kappa_{\mathrm{P}}, \alpha_{\mathrm{I}})$ within the ranges $0.83 \leq \kappa_{\mathrm{P}} \leq 1$ and $0.03 \leq \alpha_{\mathrm{I}} \leq 0.06$ (Fig. S1). Among the 1600 combinations, 683 combinations successfully achieved the induction of remission (Fig. S1 (a)), with the optimally calculated potency and duration given in Fig. S1 (b) and (c), respectively. Their calculated optimal treatment strategies for both the induction and maintenance phases are robust to the changes in the values of $(\kappa_{\mathrm{P}}, \alpha_{\mathrm{I}})$ (Fig. S2).

2 Sensitivity to weights of objective functions (k_{r}^1, k_{r}^2, k_{r}^3, k_{r}^4, k_{m}^1, k_{m}^3, k_{m}^4)

To investigate the sensitivity of the optimal treatment strategies to the weights of the objective function terms, we varied eight weights simultaneously by ±50% from their nominal values ($N = 400$): four for the induction phase (k_{r}^1, k_{r}^2, k_{r}^3, k_{r}^4) and four for the maintenance phase (k_{m}^1, k_{m}^2, k_{m}^3, k_{m}^4). We assumed $k_{\mathrm{r}}^2 = k_{\mathrm{m}}^2$ since the efficacy of the corticosteroid treatment is the same in both remission and maintenance phases. Our analysis (Fig. S2) confirmed that the calculated optimal treatment strategies were robust to the changes of the weighting parameters.

3 Sensitivity to model parameters (γ_{B}, δ_{P}, κ_{B}, γ_{R}, γ_{G}, δ_{B}, β_1, β_2, β_3)

To investigate the sensitivity of the optimal treatment strategies to the model parameters, we varied the parameters simultaneously by ±50% from their nominal values ($N = 529$). Our analysis (Fig. S2)

*r.tanaka@imperial.ac.uk

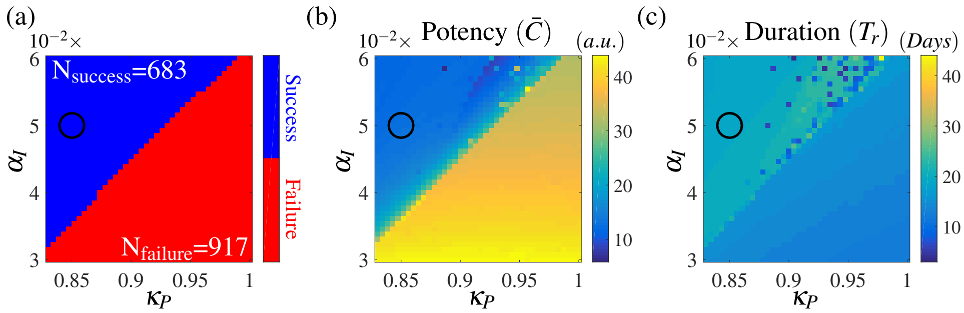

Fig. S 1: The calculated optimal treatment strategies for the induction phase for 1600 different combinations of (κ_P, α_I). (a) Binary heatmap indicating success (blue) or failure (red) to induce remission, by the calculated optimal treatment strategies; (b) The potency of corticosteroids; (c) The duration of the induction phase. The black circles represent the nominal parameter pair $(\kappa_P, \alpha_I) = (0.85, 0.05)$.

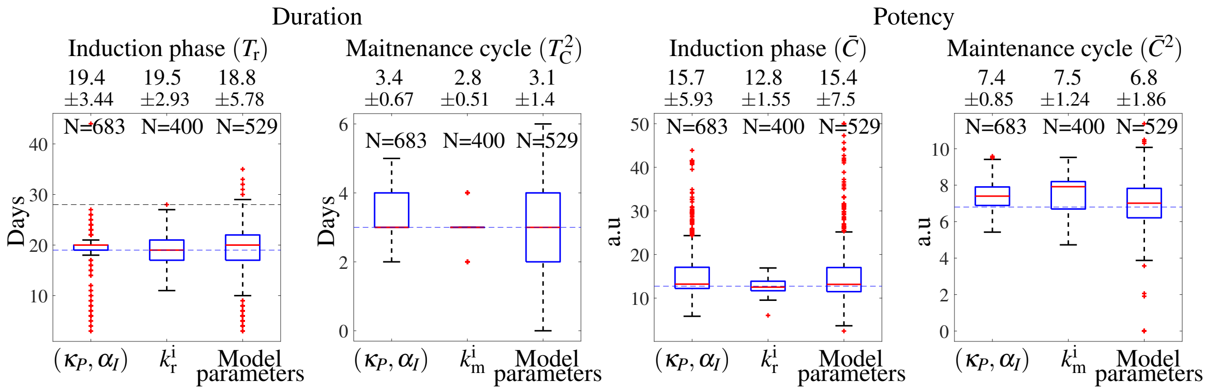

Fig. S 2: Sensitivity of the calculated optimal treatment strategies with respect to the changes in (κ_P, α_I), weights for the objective function terms (k_r^i and k_m^i), and model parameters. The blue dashed line is the result of the optimization for the nominal conditions (figure 3a). The black dashes line is the maximum acceptable induction of remission duration. The values shown are mean ± std.

confirmed that the calculated optimal treatment strategies were robust to the changes in the model parameters.
